# Supplementary material for: Biotic and abiotic controls on co-occurring nitrogen cycling processes in shallow Arctic shelf sediments
Source: Nat Commun. 2016 Oct 26;7:13145. doi: 10.1038/ncomms13145 (PMC5095177; doi:10.1038/ncomms13145)
Supplement: Supplementary Information — Supplementary Figure 1, Supplementary Tables 1-2, Supplementary Methods and Supplementary References [file ncomms13145-s1.pdf]

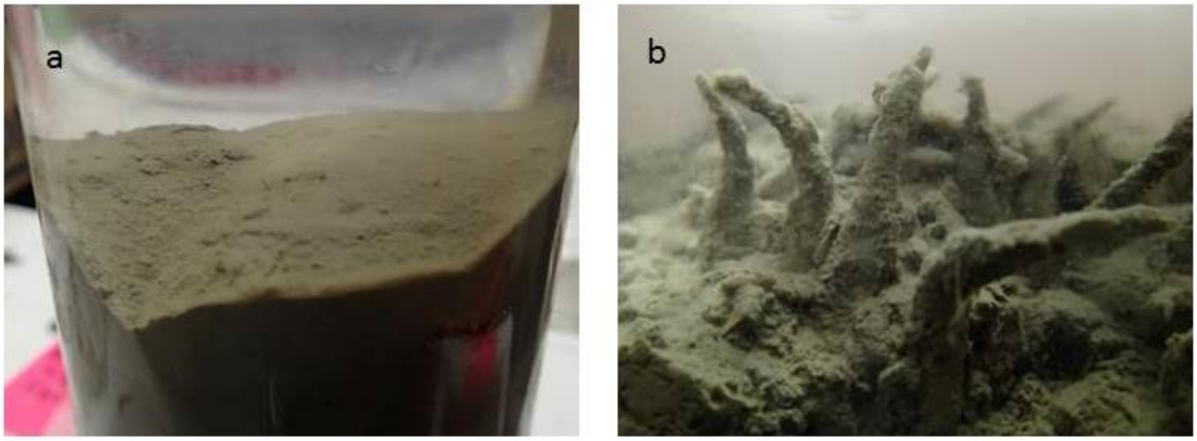

Supplementary Figure 1. Photographs of undisturbed sediment water interface in experimental cores. Cores were collected from (a) H29 and (b) CBL13. The large infauna tubes at CBL13 are occupied by the polychaete *Maldane sarsi*. Photographs taken by NDM.

Supplementary Table 1. Abundance and biomass of select fauna. Mean and s.d. (n=3) abundance (individuals m^{-2}) and biomass (grams wet weight m^{-2}) for species representing Amphipoda, Bivalvia, and Polychaeta. The three groups constituted >90% of the abundance, except for H29 where they were 83%.

	CBL11		CBL13		H17		H29		H33	
Species	Abundance	Biomass	Abundance	Biomass	Abundance	Biomass	Abundance	Biomass	Abundance	Biomass
AMPHIPODA										
Ampelisca sp.			160 ± 134	26.4 ± 12.9	10 ± 0	3.34 ± 4.40	10 ± 0	5.70 ± 6.11	20 ± 10	3.05 ± 2.56
Anonyx sp.			20 ± 0	0.67 ± 0.50	10 ± 0	0.81 ± 0.09			3.3 ± 5.77	0.06 ± 0.10
Byblis sp.	76.6 ± 28.8	2.13 ± 1.37	136. ± 86.2	11.7 ± 6.16	25 ± 21.2	1.80 ± 2.46	3.3 ± 5.77	0.16 ± 0.27	10 ± 0	1.07 ± 1.45
BIVALVIA										
Astarte borealis					20 ± 0	160 ± 23.3				
Cyclocardia crebricostata			36.6 ± 30.5	31.5 ± 11.7						
Ennucula tenuis	90 ± 10	9.00 ± 6.38	250 ± 105	23.9 ± 9.43	413. ± 92.9	44.6 ± 10.0	20 ± 14.1	1.51 ± 1.92	656 ± 134	105. ± 20.8
Liocyma fluctuosa					3.3 ± 5.77	0.14 ± 0.24	10 ± 0	8.53 ± 11.9	25 ± 21.2	22.5 ± 22.5
Macama calcarea									43.3 ± 75.0	75.9 ± 131
Macoma calcarea			6.66 ± 11.5	6.35 ± 11.0	30 ± 28.2	107 ± 124			145 ± 21.2	250. ± 15.2
Macoma loveni			3.3 ± 5.77	0.10 ± 0.18					10 ± 0	0.84 ± 0.74
Macoma moesta	120 ± 14.1	26.2 ± 4.68	25 ± 21.2	9.56 ± 8.98	56.6 ± 11.5	56.1 ± 11.8			186. ± 66.5	141. ± 17.4
Macoma sp.			20 ± 0	0.45 ± 0.01	343 ± 198	2.84 ± 2.29	30 ± 28.2	0.5 ± 0.62	443 ± 210	5.43 ± 2.18
Yoldia hyperborea	70 ± 26.4	111 ± 24.9	3.3 ± 5.77	5.03 ± 8.71	540 ± 286.	26.1 ± 11.3	76.6 ± 20.8	48.9 ± 30.4	176. ± 25.1	21.4 ± 8.51
POLYCHAETA										
Axiothella cantenata	15 ± 7.07	3.22 ± 0.19	10 ± 0	0.23 ± 0.10	20 ± 0	24.8 ± 34.8				
Cistenides hyperborea			3.3 ± 5.77	0.07 ± 0.12	16.6 ± 11.5	0.24 ± 0.22	15 ± 7.07	0.38 ± 0.08	43.3 ± 11.5	3.44 ± 2.85
Maldane sarsi			5057 ± 484	121 ± 12.5	3.3 ± 5.77	2.52 ± 4.36				
Owenia sp.									93.3 ± 64.2	3.28 ± 2.06
Spio cirrifera					6.66 ± 11.5	0.06 ± 0.10				
Sternaspis sp.	56.6 ± 15.2	4.91 ± 1.03								
Terebellides sp.	50 ± 43.5	7.17 ± 4.74	116 ± 66.5	9.38 ± 6.13	30 ± 14.1	7.55 ± 10.0	10 ± 0	1.52 ± 2.30	26.6 ± 15.2	1.23 ± 0.77

Supplementary Table 2. Surveyed literature and citations corresponding to Figure 4. Rates for denitrification (D_{14}), anammox (A_{14}), and DNRA ($DNRA_{14}$) are accompanied by standard deviation (s.d.) if provided by study. ra% is the proportion of N_2 produced by anammox out of total N_2 flux. DNRA% is the proportion of NO_3^- reduced by DNRA compared to denitrification. Study numbers correspond to references.

Temperature (°C)	D_{14} (μmol N m^{-2} h^{-1})	A_{14} (μmol N m^{-2} h^{-1})	$DNRA_{14}$ (μmol N m^{-2} h^{-1})	ra (%)	DNRA (%)	Study
5	1.8	0	4	0	68.9	1
5	9.5	0	0.5	0	5	1
5	4	0	2	0	33.3	1
9.4	5±0.5		0.5		9.0	2
9.4	10±1		0.6		5.6	2
9.4	6.5±1		0.8±0.25		10.9	2
9.4	7±1		1.5±0.5		17.6	2
9.4	8±1		0.5±0.25		5.8	2
3				7		3
11				2		3
15				0		3
16				0		3
17				0		3
21				0		3
22				0		3
3				0		3
11				2		3
15				0		3
16				0		3
17				0		3
21				5		3
22				0		3
5				21		3
6				20		3
7				20		3
11				22		3
11.5				11		3
12				12		3
15				10		3
16				13		3
17				10		3
7				15.5±5		3
8				41		3
9				13		3

11			16		3
12			20		3
13			9		3
15			17		3
16			9		3
17			21		3
18			16		3
8			10±4		3
-0.2	0.175	0.154	47		4
0.7	1	0.33	26		4
0.4	1.46	0.208	12		4
1.2	8.79	0.958	10		4
2.1	13.42	0.458	3		4
29.9			0		5
31.8			0		5
5.3			14		5
19			6		5
6.8			6.5		5
14.6	30±6				6
12.2	25±5				6
5.3	20±5				6
12.2	13±5				6
11.3	17±9				6
12.4	13±5				6
5.5	8±2				6
5.2	7±1.5				6
10.1	6±1				6
15.3	4±0.5				6
15.3	3±1				6
22.6	1±0.5				6
7.5	4.1±0.64	1.4±0.21	23		7
8.3	4.2±0.19	1.1±0.07	18		7
6.8	5±0.19	3.5±0.1	40		7
6.5	0.14±0.01	0.55±0.04	79		7
7	6.8±1.4	1.6±0.14	15		7
21	13±0.27	1.2±0.06	7		7
21	16±1.5	0.91±0.11	4		7
22			0	50	7
22			0	69.2	7
22	2.08	4.86	70		7
22			0		7
2	12.25	1.08	8		8
2	12.04	0.625	5		8

1	1.42		0.417		23		8
28.8					7.5		9
24					6.5		9
21.2					14±1		9
27.7					7.5		9
25.1					4		9
21.2					7±1		9
26.9					7		9
24.5					5		9
21.1					6		9
27.3					7		9
22.7					2		9
11.8					2		9
19.9					6		9
27.2					4		9
23.4					3		9
11.7					4		9
20					3		9
27.8					3		9
23.8					2		9
19.2					5		9
28.1					7		9
23.1					5		9
17					5		9
17.8					6		9
7.5					15±5		10
23	5.36±1.67						11
23		15.74±0.72		2.12		11.8	11
23	0.73±0.04						11
23	9.66±1.87						11
-0.5					23		12
0.5					26.7		12
-1.5					18.5		12
4					1.3		12
1.7					1.7		12
-1.3					1.8		12
-1.3					17.4		12
-1.7					34.9		12
-1.3					21.1		12
-1.7					1.4		12
0.7					10.2		12
-2.5					12		12
0					15		12

2.5				22		12
5				26		12
7.5				16		12
10				17		12
12.5				14		12
15				13		12
17.5				10		12
19				8		12
22				5		12
24				4		12
26				4		12
28				2		12
3.9	1	1.5±0.25		66		13
3.9	0.5	1		65		13
9.5	0.5	0.5		45		13
9.6	0.3	0.25		46		13
10.6	0.25	0.25		35		13
10.4	0.25	0.25		35		13
10.6	5.9±0.75	1	0.001	15	0.01	13
8.6	2.5	1	0.001	27	0.04	13
11.7	4±0.5	2.5		39		13
8.3	0.7	1.1		62		13
13.4	5±0.5	1.4	0.005	21	0.1	13
7.5	2.75±0.25	1.4	0.005	33	0.2	13
6.9	13.25±1.3	0		0		14
5.8	9.57±2.3	0		0		14
4.5	0.61±0.1	2.01±0.3		77		14
4.5	0.3±0.2	0.77±0.2		72		14

Supplementary Methods

Prior to the discovery of the anammox process, Nielsen[15] proposed adding excess $^{15}NO_3^-$ tracer to sediments and measuring denitrification of $^{15}NO_3^-$ and ambient $^{14}NO_3^-$ by the respective equations

$$D_{15} = 2 \times p30 + p29 \quad (1)$$

and

$$D_{14} = D_{15} \times r_{14} \quad (2)$$

where p29 is the production of $^{29}N_2$, p30 is the production of $^{30}N_2$, and r_{14} is the ratio of $^{14}NO_3^-$ and $^{15}NO_3^-$ undergoing nitrate reduction. As discussed by Risgaard-Petersen et al.[16], the process of anaerobic ammonium oxidation (anammox) can simultaneously produce $^{29}N_2$ from $^{15}NO_3^-$ and inflate the p29 term used to calculate denitrification. This can occur by one of two pathways with

$^{15}NO_3^-$: either unlabeled ammonium already present in porewater can combine with nitrite reduced from the added nitrate

$$^{15}NO_3^- \rightarrow {}^{15}NO_2^- + {}^{14}NH_4^+ \rightarrow {}^{29}N_2, \quad (3)$$

or added $^{15}NO_3^-$can undergo DNRA and subsequently react with already-present unlabeled nitrite

$$^{15}NO_3^- \rightarrow {}^{15}NH_4^+ + {}^{14}NO_2^- \rightarrow {}^{29}N_2. \quad (4)$$

If DNRA rates are low, as they were in the current study, the latter pathway can be disregarded. Therefore, production of $^{29}N_2$ in a given sample incubated with $^{15}NO_3^-$ ($p29_{NO3-}$) is defined as

$$p29_{NO3-} = D_{29} + A_{29}, \quad (5)$$

where D_{29} is the contribution to p29 from denitrification and A_{29} is the contribution from anammox. To accurately report denitrification using Nielsen's[15] isotope pairing technique (equations 1 and 2), the p29 from anammox must be separated from denitrification. Since anammox and denitrification are not discernable from the $p29_{NO3-}$ signal, we employed a strategy to estimate the anammox rates that would co-occur with denitrification using a $^{15}NH_4^+$ tracer. We

can measure $^{29}N_2$ produced from a sample incubated with $^{15}NH_4^+$ ($p29_{NH4+}$) by assuming the anammox process follows

$$^{15}NH_4^+ + {}^{14}NO_2^- \rightarrow {}^{29}N_2, \quad (6)$$

which is equivalent to the amount of anammox-produced N_2 from the $^{15}NH_4^+$ tracer (A_{15}); therefore,

$$A15 = p29_{NH4+}. \quad (7)$$

We assume only anammox can produce $^{29}N_2$ in this treatment. However, if coupled nitrification-denitrification also produced $^{29}N_2$ from $^{15}NH_4^+$ and ambient $^{14}NO_3^-$, then our rates of anammox presented here would be overestimated and rates of denitrification would be conservative. For the purpose of estimating maximum contribution from anammox, we assume only anammox is

responsible for $p29_{NH4+}$. Thamdrup and Dalsgaard[17] showed total anammox (A_{total}), or the amount of anammox from $^{14}NH_4^+$ and $^{15}NH_4^+$ as

$$A_{total} = p29_{NH4+} / F_A \quad (8)$$

where F_A is the proportion of $^{15}NH_4^+$ in the total NH_4^+ pool. Since

$$A_{total} = A_{15} + A_{14}, \quad (9)$$

we can then calculate A_{14}, which we refer to as the 'true' anammox that occurs without stimulus from the added $^{15}NH_4^+$, as

$$A_{14} = A_{total} – A_{15} = A_{total} – p29_{NH4+} \quad (10)$$

A_{14} is the anammox rate that can be compared to other rates like D_{14} that are standardized to the ambient ^{14}N in a sample and should not be influenced by the added ^{15}N tracer. By assuming A_{14}

in the $^{15}NH_4^+$ treatment is equivalent to A_{14} in the $^{15}NO_3^-$ treatment, we can derive A_{29} contributing to $p29_{NO3-}$ in the following steps.

Risgaard-Petersen et al.[16] showed that

$$A_{28}/A_{29} = r_{14} \tag{11}$$

where A_{28} is the $^{28}N_2$ from anammox and A_{29} is the $^{29}N_2$ from anammox. The above equation can be rearranged as

$$A_{28} = A_{29} \text{ x } r_{14}. \tag{12}$$

Since A_{14} is anammox from ambient ^{14}N, it is also defined as

$$A_{14} = 2(A_{28}) + A_{29}. \tag{13}$$

Substituting equation 12 into equation 13, we find

$$A_{14} = 2(A_{29} \text{ x } r_{14}) + A_{29}, \tag{14}$$

which can be rearranged to

$$A_{14} = A_{29}(2 \text{ x } r_{14} + 1) \tag{15}$$

so that A_{29} can be solved as

$$A_{29} = A_{14}/[(2 \text{ x } r_{14}) + 1] \quad (16)$$

since A_{14} was determined in equation 10. Finally, the newly derived A_{29} from equation 16 was used in equation 5 to solve for D_{29}, which was then used in Nielsen's[15] equations 1 and 2 to estimate denitrification without anammox confounding the p29 term.

Supplementary References

1 Bonaglia, S. *et al.* Effect of reoxygenation and Marenzelleria spp. bioturbation on Baltic Sea sediment metabolism. *Marine Ecology Progress Series* **482**, 43-55, doi:10.3354/meps10232 (2013).

2 Bonaglia, S., Nascimento, F. J., Bartoli, M., Klawonn, I. & Bruchert, V. Meiofauna increases bacterial denitrification in marine sediments. *Nature communications* **5**, 5133, doi:10.1038/ncomms6133 (2014).

3 Brin, L. D., Giblin, A. E. & Rich, J. J. Environmental controls of anammox and denitrification in southern New England estuarine and shelf sediments. *Limnology and Oceanography* **59**, 851-860, doi:10.4319/lo.2014.59.3.0851 (2014).

4 Canion, A. *et al.* Temperature response of denitrification and anaerobic ammonium oxidation rates and microbial community structure in Arctic fjord sediments. *Environmental microbiology* **16**, 3331-3344, doi:10.1111/1462-2920.12593 (2014).

5 Canion, A. *et al.* Temperature response of denitrification and anammox reveals the adaptation of microbial communities to in situ temperatures in permeable marine sediments that span 50° in latitude. *Biogeosciences* **11**, 309-320, doi:10.5194/bg-11-309-2014 (2014).

6 Deutsch, B., Forster, S., Wilhelm, M., Dippner, J. W. & Voss, M. Denitrification in sediments as a major nitrogen sink in the Baltic Sea: an extrapolation using sediment characteristics. *Biogeosciences* **7**, 3259-3271, doi:10.5194/bg-7-3259-2010 (2010).

7 Engström, P., Dalsgaard, T., Hulth, S. & Aller, R. C. Anaerobic ammonium oxidation by nitrite (anammox): Implications for N2 production in coastal marine sediments. *Geochimica et Cosmochimica Acta* **69**, 2057-2065, doi:10.1016/j.gca.2004.09.032 (2005).

8 Gihring, T. M., Lavik, G., Kuypers, M. M. & Kostka, J. E. Direct determination of nitrogen cycling rates and pathways in Arctic fjord sediments (Svalbard, Norway). *Limnology and Oceanography* **55**, 740-752 (2010).

9 Lisa, J. A., Song, B., Tobias, C. R. & Duernberger, K. A. Impacts of freshwater flushing on anammox community structure and activities in the New River Estuary, USA. *Aquatic Microbial Ecology* **72**, 17-31, doi:10.3354/ame01682 (2014).

10 Neubacher, E. C., Parker, R. E. & Trimmer, M. Short-term hypoxia alters the balance of the nitrogen cycle in coastal sediments. *Limnology and Oceanography* **56**, 651-665 (2011).

11 Porubsky, W. P., Weston, N. & Joye, S. B. Benthic metabolism and the fate of dissolved inorganic nitrogen in intertidal sediments. *Estuarine Coastal and Shelf Science* **83**, 392-402 (2009).

12 Rysgaard, S., Glud, R. N., Risgaard-Petersen, N. & Dalsgaard, T. Denitrification and anammox activity in Arctic marine sediments. *Limnology and Oceanography* **49**, 1493-1502 (2004).

13 Trimmer, M. & Nicholls, J. C. Production of nitrogen gas via anammox and denitrification in intact sediment cores along a continental shelf to slope transect in the North Atlantic. *Limnology and Oceanography* **54**, 577-589 (2009).

14 Trimmer, M., Engström, P. & Thamdrup, B. Stark contrast in denitrification and anammox across the deep Norwegian Trench in the Skagerrak. *Applied and Environmental Microbiology* **79**, 7381-7389 (2013).

15 Nielsen, L. P. Denitrification in sediment determined from nitrogen isotope pairing. *FEMS microbiology ecology* **86**, 357-362 (1992).

16 Risgaard-Petersen, N., Nielsen, L. P., Rysgaard, S., Dalsgaard, T. & Meyer, R. L. Application of the isotope pairing technique in sediments where anammox and denitrification coexist. *Limnology and Oceanography - Methods* **1**, 63-73 (2003).

17 Thamdrup, B. & Dalsgaard, T. Production of N_2 through anaerobic ammonium oxidation coupled to nitrate reduction in marine sediments. *Applied and Environmental Microbiology* **68**, 1312-1318 (2002).
